# The association between social frailty, psychological resilience, and subsequent cognitive outcomes in older adults: A prospective cohort study

**DOI:** 10.1016/j.jnha.2025.100576

**Published:** 2025-05-06

**Authors:** Yun-Jing Zhang, Cong Zhang, Qi-Yuan Lyu

**Affiliations:** School of Nursing, Jinan University, Guangzhou, China

**Keywords:** Social frailty, Cognitive function, Older adults, Psychological resilience, Cognitive impairment, Greater cognitive decline

## Abstract

**Background:**

The associations among social frailty, psychological resilience, and cognitive function, as well as their variations across sex and age, remain to be explored. The objective of the present study was to investigate these complex relationships in older adults.

**Methods:**

This study included 5555 participants from the CLHLS and categorized them into socially robust (n = 2229; 40.12%), social pre-frailty (n = 2624; 47.24%), and social frailty (n = 702; 12.64%) groups. The relationship between social frailty and cognitive outcomes was analyzed using logistic regression models and restricted cubic splines. The combined effects of social frailty and psychological resilience on cognitive outcomes were also analyzed. The mediating role of psychological resilience was evaluated using the SPSS PROCESS macro program.

**Results:**

Social pre-frailty (odds ratio [OR]: 1.81, 95%CI: 1.48–2.21) and social frailty (OR: 2.40, 95%CI: 1.87–3.09) were positively associated with cognitive impairment. Furthermore, social pre-frailty (OR: 1.71, 95%CI: 1.47–2.00), and social frailty (OR: 2.10, 95%CI: 1.69–2.60) were also positively associated with greater cognitive decline. Adverse cognitive outcomes demonstrated a nonlinear relationship with social frailty. Compared to individuals with social robustness and high psychological resilience, those with social frailty and low psychological resilience demonstrated higher odds ratios of developing cognitive impairment (OR: 3.65, 95%CI: 2.61–5.10) and experiencing greater cognitive decline (OR: 3.05; 95%CI: 2.33–4.00). The relationship between social frailty and negative cognitive outcomes was more pronounced among women and individuals younger than 80 years and exhibited a nonlinear pattern. Psychological resilience mediated the relationship between social frailty and cognitive outcomes in men (β = −0.0091, *P* < 0.05) and individuals with advanced age (β = −0.0087, *P* < 0.05).

**Conclusions:**

Social frailty is an independent influencing factor of adverse cognitive outcomes. Psychological resilience mediates the relationship between social frailty and cognitive function in men and subjects of advanced age.

## Highlights

• Social frailty (SF) raises odds ratios of two cognitive outcomes in older adults.
• SF is more closely linked to cognitive impairment than to greater cognitive decline.
• Older adults with SF and low resilience are most prone to adverse cognition.
• Resilience mediates the association between SF and cognitive function.
• These associations exhibit sex and age differences.

## 1 Introduction

Cognitive impairment is characterized by declining memory, attention, and cognitive function, and encompasses the entire spectrum from mild cognitive impairment (MCI) to dementia, thereby affecting the quality of life in older adults [[1], [2], [3]]. Mild cognitive impairment and cognitive decline are recognized as transitional stages between normal aging and dementia, closely linked to health and well-being in later life [1,4]. The current global prevalence of MCI is between 6% to 12% [5], and approximately 39.2% of individuals with MCI are projected to progress to dementia [6].

Social frailty (SF) is one of the most complex subdomains of frailty but has not been studied in detail [7]. Social frailty (SF) is defined as the loss or potential loss of social resources, behaviors, activities, and self-management capabilities required to meet basic social needs throughout the life course [8]. The proportion of older adults affected by social frailty ranges from 7.4% to 22% globally, and 4.9% to 17% in China [[9], [10], [11], [12]]. Social frailty is associated with elevated risk of mortality, disability, and depression [13]. Furthermore, cognitive impairment and Alzheimer's disease are more common among older persons with social frailty, and the impact of social frailty on cognition extends beyond typical age-related decline [14,15]. This association may involve inflammatory mechanisms. Social isolation and social support are often considered as components of social frailty [10] and are linked with inflammatory markers [[16], [17], [18]]. Inflammation modulates the development and progression of cognitive impairment and dementia by suppressing angiogenesis and neurotrophic factor function, and enhancing neuronal apoptosis and myelin structural damage [10,19,20].

Psychological resilience is a dynamic process and a protective mechanism against negative stressors [21]. It promotes recovery from stress or adversity and enhances disease resistance, adaptation, and personal growth [22]. Psychological and social factors are significantly linked to dementia prevention [23]. The relationship between social support and cognitive function is mediated by psychological resilience [24]. Therefore, psychological resilience may serve as a mediator of the relationship between social frailty and cognitive function.

Cognitive impairment in older adults is a major public health concern, especially with the accelerating global aging population. Social frailty is a modifiable risk factor associated with adverse cognitive outcomes, but the underlying mechanisms of this relationship are not fully elucidated. It is not known whether the association between social frailty and cognitive outcomes is linear or nonlinear. Moreover, it is not known whether psychological resilience mediates the association between social frailty and cognitive outcomes. The heterogeneity of this relationship across different age and sex groups remains to be evaluated. These issues have limited the development and implementation of effective and targeted prevention strategies that can preserve and enhance cognitive function in older adults and improve their quality of life. China has the largest number of patients with dementia worldwide [25,26]. Moreover, China’s unique socio-cultural context offers a valuable backdrop for examining these associations. Therefore, in this study, we used the CLHLS to investigate the association between social frailty, psychological resilience, and cognitive outcomes, considering sex and age differences to provide novel insights for dementia prevention.

## 2 Methods

### 2.1 Study design and participants

The approval for CLHLS (IRB00001052-13074) was provided by the Biomedical Ethics Committee of Peking University and has been described in a previous report [27]. This study used data from two non-overlapping cohorts (2008–2011/2012 and 2011/2012–2014) with an average follow-up duration of 2.98 years. Exclusion criteria included missing data on social frailty, psychological resilience, confounders and MMSE scores, cognitive impairment at baseline, death and loss to follow-up during the study period, and participants younger than 60 years. Finally, we included 5555 individuals in this study (Figure A1).

### 2.2 Cognitive outcomes

The Mini-Mental State Examination (MMSE) was composed of 24 items across the following five domains: orientation, registration, attention and calculation, recall, and language. The total score ranged from 0 to 30. In this study, a total MMSE score below 24 indicated cognitive impairment [4]. Furthermore, greater cognitive decline was defined as a decline of more than 10% in MMSE scores during the follow-up periods when calculated using the following formula: (baseline MMSE-follow-up MMSE)/baseline MMSE × 100% [28].

### 2.3 Social frailty

Guided by Bunt’s conceptual framework and relevant studies, the study was adapted to the context of the CLHLS, ultimately including six items. Items related to living arrangements, economic status, and social participation were based on previous studies by Yamada et al. [29], Nagai et al. [30], and Lou et al. [4]. Items regarding education and marital status were based on the study by Lee et al. [31]. Items for social support were derived from studies by Pek et al. [9], Lee et al. [31], and Fan et al. [28]. The distribution of six social frailty items used in this study, which were based on Bunt's conceptual framework is shown in Figure A2 and Table A1. Economic status was assessed by asking participants "Do you have enough money to meet your daily needs?" with response options "yes" or "no." Those who answered "no" were classified as lacking sufficient financial resources. Participants received 1 point each for the following responses: living alone; unmarried; no formal education; and insufficient financial means. Social participation included the following three activities: playing mahjong; participating in organized social activities; and participating in outings. Older adults who did not engage in any of these activities were assigned 1 point. This study assessed social support using the following four questions: (1) Who do you talk to most often? (2) If you have something on your mind, who do you speak to? (3) If you face a problem or difficulty, who do you seek help from? (4) Currently, who mainly cares for you when you are unwell or sick? One point was awarded for answering "nobody" to any of these questions.

The total score for social frailty was 6 points. Participants in this study were classified into the following three groups: socially robust (0–1 points), social pre-frailty (2–3 points), and social frailty (4–6 points). Based on the results, this study classified 2229 participants (40.12%) in the socially robust group, 2624 participants (47.24%) in the social pre-frailty group, and 702 participants (12.64%) in the social frailty group. This distribution pattern was similar to previous studies conducted in China [32] and Korea [33]. The distribution of baseline characteristics, including residence, self-reported health, exercise, cognitive outcomes, and psychological resilience, across the three social frailty subgroups supported the selection of cut-off values [14,31].

### 2.4 Psychological resilience

Based on a previous study [4], the psychological resilience of the participants was assessed with the following five-item questionnaire: (1) See the bright side (optimism); (2) Make your own choices (autonomy); (3) Feeling less useful as one gets older (age-related decline); (4) Feeling nervous or scared (anxiety); and (5) Feeling lonely (loneliness). Each item was rated on a 5-point scale. Higher scores indicated greater psychological resilience. Based on the median score, subjects were classified into high and low psychological resilience groups [4].

### 2.5 Covariates

The covariates included socio-demographics, lifestyle, and health status. Socio-demographic factors included age (<80 y or ≥80 y), sex (males or females), and residence (urban or rural). Lifestyle factors included smoking (never, current, or former), drinking (never, current, or former), and exercise (never, current, or former). Health status factors included body mass index (BMI, 18.5−23.9, ≤18.4, ≥24), self-reported health (good, so-so, or bad), activities of daily living (ADL, normal or limited), sleep quality (good, so-so, or bad), MMSE score at baseline, hypertension, cerebrovascular disease, and diabetes (yes or no).

### 2.6 Statistical analysis

Descriptive statistical analysis was performed to compare differences between the socially robust, social pre-frailty, and social frailty groups. The associations between social frailty and cognitive outcomes were evaluated using logistic regression models. Model 1 was adjusted for age, sex, and residence. Model 2 was adjusted for smoking, drinking, exercise, BMI, ADL, sleep quality, self-reported health, MMSE score at baseline, hypertension, diabetes, and cerebrovascular disease in addition to parameters adjusted in Model 1. Model 3 was adjusted for the psychological resilience score and all parameters adjusted in Model 2.

Next, we analyzed potential nonlinear links between social frailty and negative cognitive outcomes using restricted cubic splines (RCS) with the "rms" R package. RCS is a nonparametric modeling technique that uses spline functions to model continuous variables. They are piecewise cubic polynomials that ensure continuity and second-order differentiability at the knots [34]. To determine the ideal number of knots, we built RCS models with three to five knots and chose the model with the least Akaike Information Criterion (AIC). A nonlinear association between variables was considered present when both the overall and nonlinear p-values were less than 0.05 [35].

Spearman correlation analysis was used to evaluate the associations between cognitive functions, psychological resilience, and social frailty. The mediating effect of psychological resilience was tested using the SPSS PROCESS macro program in the SPSS software. Finally, we examined the combined effects of social frailty and psychological resilience on adverse cognitive outcomes. Apart from the joint analysis, where sex and age differences were not evaluated because of small sample sizes in certain subcategories, all the other analyses were further stratified by sex and age. All statistical analyses were performed in SPSS 26.0 and R version 4.3.3 software. A two-tailed *P* < 0.05 was considered statistically significant.

Sensitivity analyses were performed to evaluate the reliability of our findings. BMI, MMSE score at baseline, and psychological resilience scores were first standardized using z-scores. Then, additional covariates were added to the models for subsequent analyses. Finally, multiple imputations were performed on the covariates to examine the results.

## 3 Results

### 3.1 Baseline characteristics of participants

This study included 5555 subjects with an average follow-up duration of 2.98 years **(**Table 1**)**. In 2008, 16954 individuals were surveyed, but during follow-up, 8536 (50.35%) subjects were lost by 2011/2012. Some participants with follow-up records were excluded due to not meeting the inclusion criteria or having missing values for key variables. Therefore, 4673 participants were included from the 2008–2011/2012 cohort and accounted for 55.51% of those successfully followed up. After excluding participants from the 2008–2011/2012 cohort, 5092 individuals participated in the 2011/2012 survey. Subsequently, 2483 (48.76%) participants were lost to follow-up by 2014. Eventually, 882 participants were included from the 2011/2012–2014 cohort and represented 33.81% of those successfully followed up.

**Table 1:** Baseline characteristics of the included participants.

Characteristics	Overall (n = 5555)	Robust (n = 2229)	Social pre-frailty (n = 2624)	Social frailty (n = 702)	*P*
Age (%)					**<0.001**
<80 y	3101 (55.82%)	1614 (72.41%)	1217 (46.38%)	270 (38.46%)	
≥80 y	2454 (44.18%)	615 (27.59%)	1407 (53.62%)	432 (61.54%)	
Sex (%)					**<0.001**
Males	2908 (52.35%)	1617 (72.54%)	1095 (41.73%)	196 (27.92%)	
Females	2647 (47.65%)	612 (27.46%)	1529 (58.27%)	506 (72.08%)	
Residence (%)					**<0.001**
Urban	2127 (38.29%)	1008 (45.22%)	928 (35.37%)	191 (27.21%)	
Rural	3428 (61.71%)	1221 (54.78%)	1696 (64.63%)	511 (72.79%)	
BMI (%)					**<0.001**
18.5−23.9	3199 (57.59%)	1310 (58.77%)	1506 (57.39%)	383 (54.56%)	
≤18.4	1200 (21.60%)	340 (15.25%)	652 (24.85%)	208 (29.63%)	
≥24	1156 (20.81%)	579 (25.98%)	466 (17.76%)	111 (15.81%)	
Smoking (%)					**<0.001**
Never	3351 (60.32%)	1056 (47.38%)	1761 (67.11%)	534 (76.07%)	
Current	1360 (24.48%)	730 (32.75%)	526 (20.05%)	104 (14.81%)	
Former	844 (15.19%)	443 (19.87%)	337 (12.84%)	64 (9.12%)	
Drinking (%)					**<0.001**
Never	3621 (65.18%)	1230 (55.18%)	1851 (70.54%)	540 (76.92%)	
Current	1219 (21.94%)	664 (29.79%)	453 (17.26%)	102 (14.53%)	
Former	715 (12.87%)	335 (15.03%)	320 (12.20%)	60 (8.55%)	
Exercise (%)					**<0.001**
Never	2979 (53.63%)	999 (44.82%)	1528 (58.23%)	452 (64.39%)	
Current	2099 (37.79%)	1047 (46.97%)	861 (32.81%)	191 (27.21%)	
Former	477 (8.59%)	183 (8.21%)	235 (8.96%)	59 (8.40%)	
Self-reported health (%)					**<0.001**
Good	3110 (55.99%)	1353 (60.70%)	1445 (55.07%)	312 (44.44%)	
So-so	1792 (32.26%)	671 (30.10%)	865 (32.96%)	256 (36.47%)	
Bad	653 (11.76%)	205 (9.20%)	314 (11.97%)	134 (19.09%)	
ADL (%)					**<0.001**
Normal	5353 (96.36%)	2181 (97.85%)	2489 (94.86%)	683 (97.29%)	
Limited	202 (3.64%)	48 (2.15%)	135 (5.14%)	19 (2.71%)	
Sleep quality (%)					**<0.001**
Good	3751 (67.52%)	1609 (72.18%)	1753 (66.81%)	389 (55.41%)	
So-so	1259 (22.66%)	441 (19.78%)	617 (23.51%)	201 (28.63%)	
Bad	545 (9.81%)	179 (8.03%)	254 (9.68%)	112 (15.95%)	
Hypertension (%)					0.513
No	4260 (76.69%)	1719 (77.12%)	1995 (76.03%)	546 (77.78%)	
Yes	1295 (23.31%)	510 (22.88%)	629 (23.97%)	156 (22.22%)	
Diabetes (%)					**<0.001**
No	5367 (96.62%)	2130 (95.56%)	2549 (97.14%)	688 (98.01%)	
Yes	188 (3.38%)	99 (4.44%)	75 (2.86%)	14 (1.99%)	
Cerebrovascular disease (%)					0.100
No	5257 (94.64%)	2093 (93.90%)	2492 (94.97%)	672 (95.73%)	
Yes	298 (5.36%)	136 (6.10%)	132 (5.03%)	30 (4.27%)	
Cognitive impairment (%)					**<0.001**
No	4516 (81.30%)	2051 (92.01%)	1999 (76.18%)	466 (66.38%)	
Yes	1039 (18.70%)	178 (7.99%)	625 (23.82%)	236 (33.62%)	
Greater cognitive decline (%)					**<0.001**
No	3971 (71.49%)	1852 (83.09%)	1715 (65.36%)	404 (57.55%)	
Yes	1584 (28.51%)	377 (16.91%)	909 (34.64%)	298 (42.45%)	
Psychological resilience (%)					**<0.001**
High	2162 (38.92%)	1136 (50.96%)	873 (33.27%)	153 (21.79%)	
Low	3393 (61.08%)	1093 (49.04%)	1751 (66.73%)	549 (78.21%)	
Education					**<0.001**
Literate	2934 (52.82%)	1941 (87.08%)	904 (34.45%)	89 (12.68%)	
Illiterate	2621 (47.18%)	288 (12.92%)	1720 (65.55%)	613 (87.32%)	
Living alone					**<0.001**
No	4637 (83.47%)	2214 (99.33%)	2236 (85.21%)	187 (26.64%)	
Yes	918 (16.53%)	15 (0.67%)	388 (14.79%)	515 (73.36%)	
Marital status					**<0.001**
With a spouse	3048 (54.87%)	1997 (89.59%)	1033 (39.37%)	18 (2.56%)	
Without a spouse	2507 (45.13%)	232 (10.41%)	1591 (60.63%)	684 (97.44%)	
Insufficient financial resources					**<0.001**
No	4512 (81.22%)	2116 (94.93%)	2066 (78.73%)	330 (47.01%)	
Yes	1043 (18.78%)	113 (5.07%)	558 (21.27%)	372 (52.99%)	
Social participation					**<0.001**
Yes	2171 (39.08%)	1504 (67.47%)	616 (23.48%)	51 (7.26%)	
No	3384 (60.92%)	725 (32.53%)	2008 (76.52%)	651 (92.74%)	
Social support					**<0.001**
Yes	5230 (94.15%)	2209 (99.10%)	2496 (95.12%)	525 (74.79%)	
No	325 (5.85%)	20 (0.90%)	128 (4.88%)	177 (25.21%)	
MMSE score at baseline, median [25th, 75th]	29.00 [27.00, 30.00]	29.00 [28.00, 30.00]	28.50 [27.00, 29.00]	28.00 [26.00, 29.00]	**<0.001**
Psychological resilience, median [25th, 75th]	20.00 [18.00, 22.00]	21.00 [19.00, 23.00]	19.00 [17.00, 21.00]	18.00 [16.00, 20.00]	**<0.001**

Abbreviations: BMI, Body Mass Index; ADL, Activities of Daily Living; MMSE, the Mini-Mental State Examination.

*P*-values were calculated using chi-square tests or rank sum tests.

Among the 5555 participants, 2908 (52.35%) were males and 2647 (47.65%) were females. At baseline, 2624 participants (47.24%) were categorized in the social pre-frailty group and 702 (12.64%) were included in the social frailty group. Between-group comparisons differed in age, sex, residence, smoking, drinking, exercise, BMI, ADL, sleep quality, self-reported health, MMSE score at baseline, diabetes, and psychological resilience.

### 3.2 Association between social frailty and cognitive outcomes

Social pre-frailty (OR: 1.81, 95%CI: 1.48–2.21) and social frailty (OR: 2.40, 95%CI: 1.87–3.09) were positively associated with subsequent cognitive impairment. Furthermore, social pre-frailty (OR: 1.71, 95%CI: 1.47–2.00) and social frailty (OR: 2.10, 95%CI: 1.69–2.60) were positively associated with greater cognitive decline. After stratification by sex and age, social pre-frailty and social frailty were more strongly linked with negative cognitive outcomes in females and individuals aged 60–79 years (Table 2).

**Table 2:** Association of social frailty with subsequent cognitive outcomes.

	Model 1 OR (95%CI)	Model 2 OR (95%CI)	Model 3 OR (95%CI)
**Cognitive impairment**			
**Total**			
Robust	Ref.	Ref.	Ref.
Social pre-frailty	2.13 (1.76, 2.58)	1.86 (1.53, 2.27)	1.81 (1.48, 2.21)
Social frailty	2.95 (2.31, 3.75)	2.56 (2.00, 3.29)	2.40 (1.87, 3.09)
*P* for trend	<0.001	<0.001	<0.001
**Sex**			
**Males**			
Robust	Ref.	Ref.	Ref.
Social pre-frailty	2.06 (1.60, 2.65)	1.85 (1.42, 2.40)	1.77 (1.36, 2.30)
Social frailty	2.82 (1.90, 4.17)	2.49 (1.65, 3.75)	2.19 (1.44, 3.33)
*P* for trend	<0.001	<0.001	<0.001
**Females**			
Robust	Ref.	Ref.	Ref.
Social pre-frailty	2.24 (1.66, 3.04)	1.91 (1.40, 2.61)	1.87 (1.37, 2.56)
Social frailty	3.11 (2.21, 4.38)	2.60 (1.83, 3.70)	2.49 (1.75, 3.55)
*P* for trend	<0.001	<0.001	<0.001
**Age**			
**< 80 y**			
Robust	Ref.	Ref.	Ref.
Social pre-frailty	2.50 (1.83, 3.40)	2.34 (1.71, 3.21)	2.31 (1.68, 3.18)
Social frailty	3.73 (2.47, 5.62)	3.57 (2.34, 5.45)	3.45 (2.24, 5.29)
*P* for trend	<0.001	<0.001	<0.001
**≥ 80 y**			
Robust	Ref.	Ref.	Ref.
Social pre-frailty	1.89 (1.48, 2.42)	1.62 (1.26, 2.09)	1.56 (1.21, 2.02)
Social frailty	2.55 (1.89, 3.45)	2.14 (1.56, 2.92)	1.99 (1.45, 2.73)
*P* for trend	<0.001	<0.001	<0.001
**Greater cognitive decline**			
**Total**			
Robust	Ref.	Ref.	Ref.
Social pre-frailty	1.70 (1.46, 1.97)	1.76 (1.50, 2.05)	1.71 (1.47, 2.00)
Social frailty	2.04 (1.66, 2.51)	2.21 (1.79, 2.74)	2.10 (1.69, 2.60)
*P* for trend	<0.001	<0.001	<0.001
**Sex**			
**Males**			
Robust	Ref.	Ref.	Ref.
Social pre-frailty	1.69 (1.39, 2.05)	1.77 (1.45, 2.17)	1.72 (1.40, 2.10)
Social frailty	1.53 (1.08, 2.16)	1.67 (1.16, 2.39)	1.51 (1.05, 2.19)
*P* for trend	<0.001	<0.001	<0.001
**Females**			
Robust	Ref.	Ref.	Ref.
Social pre-frailty	1.78 (1.40, 2.27)	1.82 (1.42, 2.34)	1.79 (1.40, 2.29)
Social frailty	2.39 (1.80, 3.18)	2.54 (1.89, 3.41)	2.44 (1.81, 3.28)
*P* for trend	<0.001	<0.001	<0.001
**Age**			
**< 80 y**			
Robust	Ref.	Ref.	Ref.
Social pre-frailty	1.82 (1.47, 2.25)	1.98 (1.59, 2.48)	1.95 (1.56, 2.44)
Social frailty	2.33 (1.69, 3.21)	2.67 (1.91, 3.72)	2.56 (1.83, 3.59)
*P* for trend	<0.001	<0.001	<0.001
**≥ 80 y**			
Robust	Ref.	Ref.	Ref.
Social pre-frailty	1.56 (1.26, 1.93)	1.58 (1.27, 1.97)	1.53 (1.23, 1.91)
Social frailty	1.83 (1.39, 2.41)	1.94 (1.46, 2.59)	1.83 (1.37, 2.45)
*P* for trend	<0.001	<0.001	<0.001

Model 1 adjusted for age, sex, and residence.

Model 2 adjusted for all factors adjusted in model 1 as well as smoking, drinking, exercise, BMI, ADL, sleep quality, self-reported health, MMSE score at baseline, hypertension, diabetes, and cerebrovascular disease.

Model 3 adjusted for all factors adjusted in model 2 plus psychological resilience score.

### 3.3 Assessing longitudinal relationship between social frailty and cognitive outcomes using RCS

**Fig. 1:**
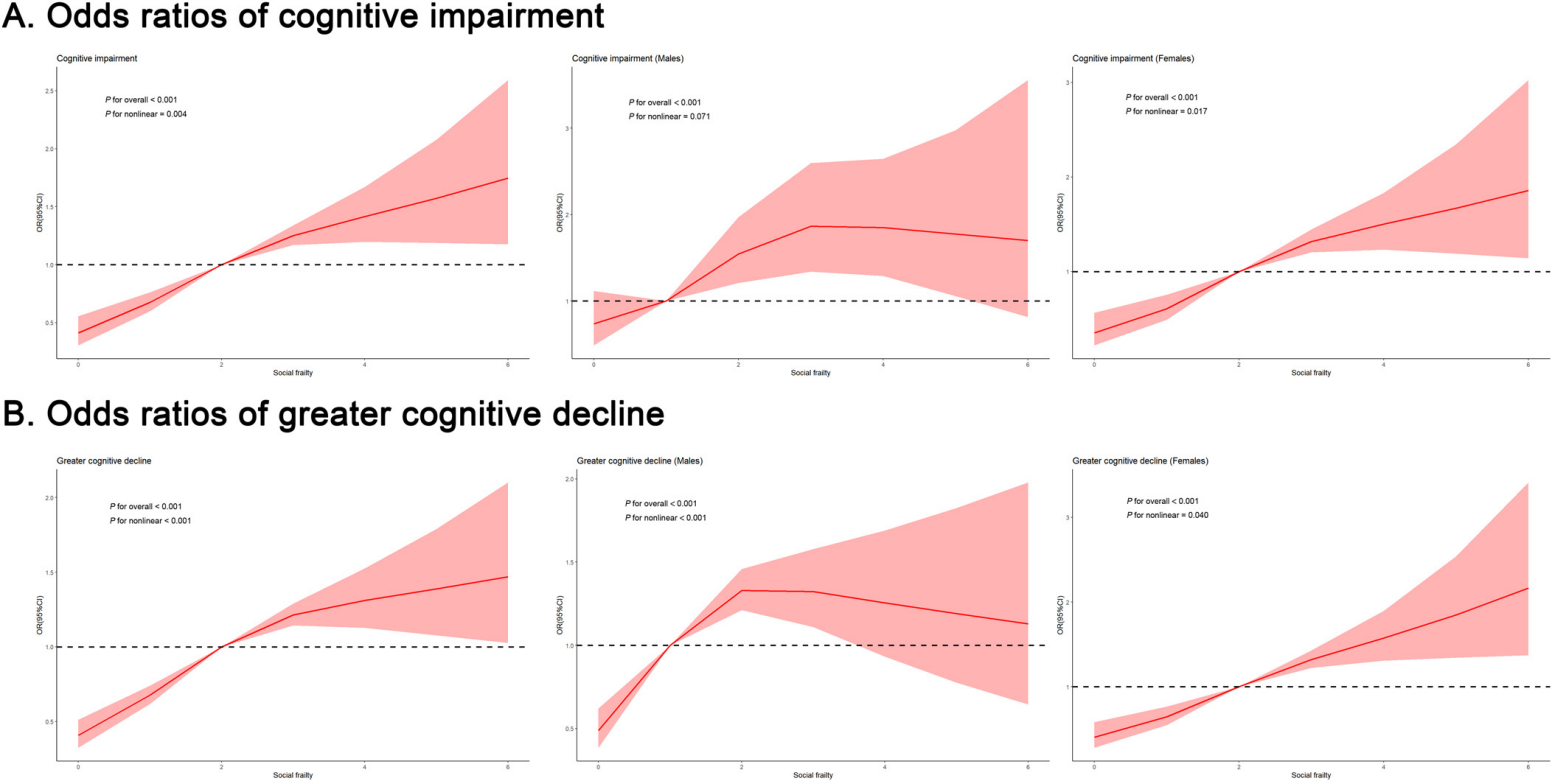
Restricted cubic spline regression model results show the association between social frailty and cognitive outcomes in the entire cohort as well as female and male subgroups. Abbreviation: OR, Odds ratio. All models adjusted for age, sex, residence, smoking, drinking, exercise, BMI, ADL, sleep quality, self-reported health, psychological resilience score, MMSE score at baseline, hypertension, diabetes, and cerebrovascular disease.

**Fig. 2:**
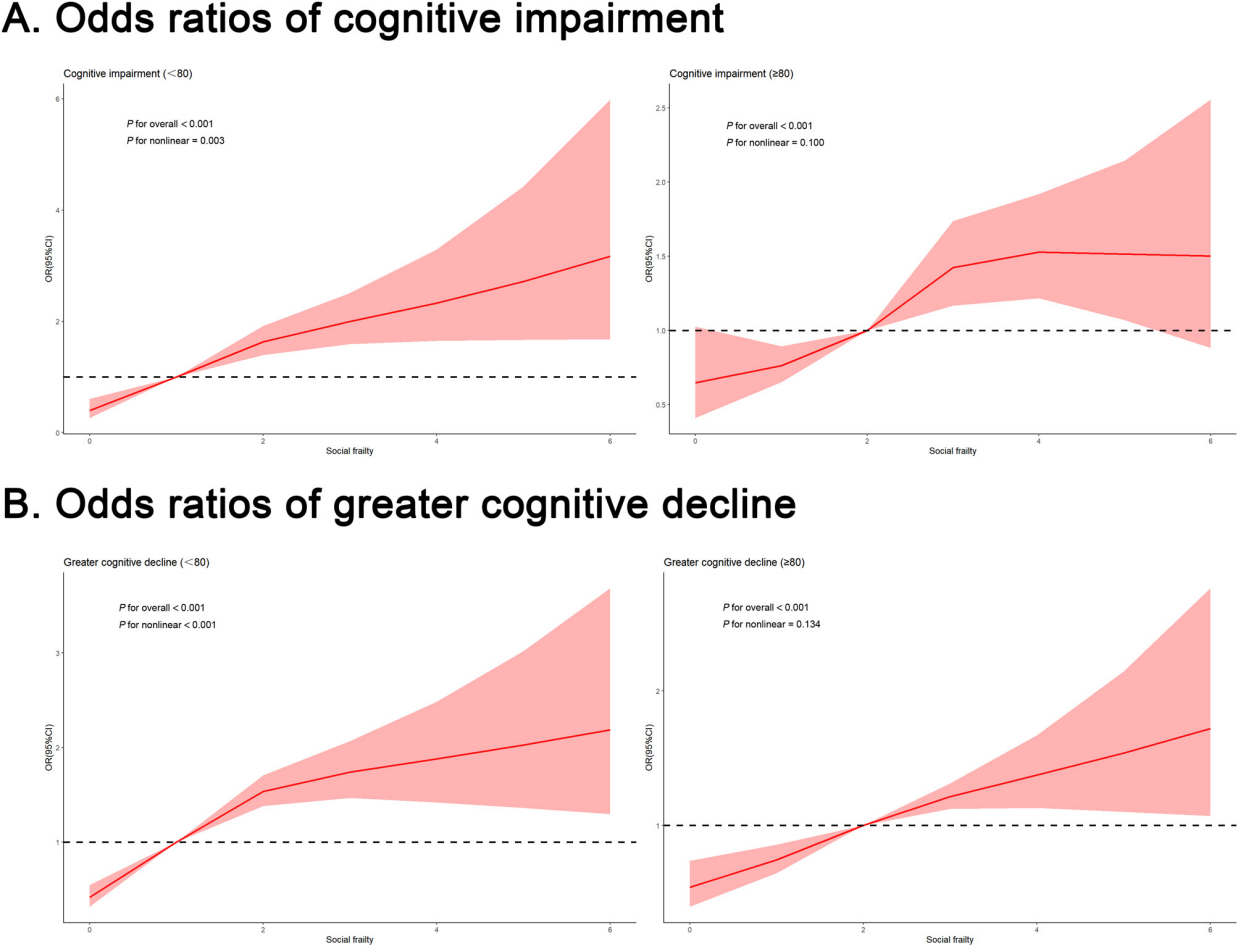
Restricted cubic spline regression model results show the association between social frailty and cognitive outcomes in subjects belonging to >80 y and <80 y subgroups. Abbreviation: OR, Odds ratio All models adjusted for age, sex, residence, smoking, drinking, exercise, BMI, ADL, sleep quality, self-reported health, psychological resilience score, MMSE score at baseline, hypertension, diabetes, and cerebrovascular disease.

Fig. 1, Fig. 2 show the results of logistic regression with an RCS to investigate the longitudinal relationship between social frailty and cognitive outcomes. Social frailty demonstrated a nonlinear association with cognitive outcomes in both the overall population and females (*P* for overall < 0.001; *P* for nonlinear <0.05). Furthermore, social frailty showed a nonlinear association with greater cognitive decline in males (*P* for overall <0.001; *P* for nonlinear <0.001). When stratified by age, social frailty demonstrated a nonlinear relationship with two cognitive outcomes in the 60∼79-year-old subgroup (*P* for overall <0.001; *P* for nonlinear <0.05).

### 3.4 Mediating effect analyses

Spearman correlation coefficients for the relationships between social frailty, psychological resilience, and MMSE score are shown in Table A2. The results of stepwise regression are shown in Table A3.

Table 3 shows the results of the Bootstrap method with 5000 resampling iterations for testing the mediating effects. Our data showed that psychological resilience partially mediated the association between social frailty and cognitive function in the overall population, males, and older individuals aged ≥80 years. In the overall population, the total effect of social frailty on cognitive function was −0.1308, including an indirect effect of −0.0064, which accounted for 4.89% of the total effect. Among men, the total effect of social frailty on cognitive function was −0.1221, including an indirect effect of −0.0091, which accounted for 7.45% of the total effect. In older individuals aged 80 years or above, the total effect of social frailty on cognitive function was −0.1289, including an indirect effect of −0.0087, which accounted for 6.75% of the total effect.

**Table 3:** Mediation effects of psychological resilience on the association between social frailty and cognitive function.

Effect	Effect value	SE	BootLLCI	BootULCI
**Total**				
Total effect	−0.1308	0.0143	−0.1587	−0.1028
Direct effect	−0.1244	0.0145	−0.1527	−0.0961
Mediating effect	−0.0064	0.0024	−0.0113	−0.0014
**Males**				
Total effect	−0.1221	0.0191	−0.1594	−0.0847
Direct effect	−0.1130	0.0193	−0.1509	−0.0751
Mediating effect	−0.0091	0.0035	−0.0164	−0.0026
**Females**				
Total effect	−0.1260	0.0195	−0.1641	−0.0878
Direct effect	−0.1211	0.0197	−0.1597	−0.0825
Mediating effect	−0.0048	0.0033	−0.0115	0.0015
**<80 y**				
Total effect	−0.1617	0.0193	−0.1995	−0.1239
Direct effect	−0.1599	0.0196	−0.1984	−0.1215
Mediating effect	−0.0018	0.0039	−0.0094	0.0059
**≥80 y**				
Total effect	−0.1289	0.0219	−0.1718	−0.0859
Direct effect	−0.1202	0.0221	−0.1635	−0.0769
Mediating effect	−0.0087	0.0034	−0.0156	−0.0025

Abbreviation: Effect value, standardized regression coefficient; SE, standard error; BootLLCI, bootstrapping lower limit confidence interval; BootULCI, bootstrapping upper limit confidence interval.

All models were adjusted for age, sex, residence, smoking, drinking, exercise, BMI, ADL, sleep quality, self-reported health, MMSE score at baseline, hypertension, diabetes, and cerebrovascular disease.

### 3.5 Joint analyses

Individuals with social frailty and low psychological resilience had odds ratios of 3.65 (95% CI: 2.61–5.10) for developing cognitive impairment and 3.05 (95% CI: 2.33–4.00) for experiencing greater cognitive decline compared to those who were socially robust and had high psychological resilience (Table A4).

### 3.6 Sensitivity analysis

Sensitivity analyses confirmed the robustness of our findings (Table A5-A12 in Appendix 1).

## 4 Discussion

This cohort study established that social frailty was associated with adverse cognitive outcomes and exhibited a nonlinear dose-response relationship. Social frailty shows a stronger association with cognitive impairment compared to greater cognitive decline. Older individuals who are socially pre-frail and socially frail had odds ratios of 1.81 and 2.40, respectively, for developing cognitive impairment, and 1.71 and 2.10, respectively, for experiencing greater cognitive decline, compared to those who are socially robust. The odds ratios for adverse cognitive outcomes were highest for older adults experiencing both social frailty and low psychological resilience. Compared to those with social robustness and high psychological resilience, individuals with social frailty and low psychological resilience showed odds ratios of 3.65 for cognitive impairment and 3.05 for greater cognitive decline. Furthermore, psychological resilience played a partial mediating role in the association between social frailty and cognitive function, accounting for 4.89% of the total mediating effect.

The relationship between social frailty and negative cognitive outcomes was more pronounced in women and adults under 80 years of age and exhibited a nonlinear dose-response relationship. Psychological resilience partially mediated the relationship between social frailty and cognitive function among men and individuals aged 80 years or older.

The factors used to assess social frailty in this study were based on Bunt's conceptual framework and factors reported by previous studies. However, some differences remain in the specific components of social frailty compared to prior studies. For example, studies by Lee et al. [31] and Tsutsumimoto et al. [36] evaluated factors such as a sense of usefulness and helpful behavior. However, this study did not assess self-worth because relevant items were not available in the CLHLS, and concerns about social desirability bias may have resulted in underreporting [9]. This study included education as a factor in the assessment of social frailty based on previous studies [37] and taking into account specific characteristics of the Chinese socio-cultural environment. Educational opportunities were fewer for older women compared to older men, partly because of traditional factors. Furthermore, disparities in economic development may contribute to unequal access to education [38]. Lower levels of education among older adults not only impacts their cognitive reserve [39], but also hinders their social participation and access to resources, thereby exacerbating the digital divide and increasing the risk of social frailty [38]. In conclusion, education is a useful indicator for assessing the influence of traditional sex norms and regional disparities in China and contributes towards a more accurate assessment of social frailty among older adults. In this study, the distribution of social frailty subgroups was consistent with previous findings [32,33]. However, the questionnaire design contributed to a higher prevalence of social frailty and a stronger link with cognitive impairment among women than observed in earlier studies [10,14,15].

Lee et al. [14] reported that the persistent impact of social frailty on cognitive decline in older adults exceeded the range of age-related deterioration. Several studies conducted in China and Japan have shown that social frailty negatively affects cognitive function and is associated with cognitive frailty and Alzheimer's disease [15,40,41]. This study demonstrated that social frailty was linked to adverse cognitive outcomes. Therefore, our findings aligned with previously reported findings. A large-sample cohort study demonstrated that the association between social frailty and motoric cognitive risk syndrome was more pronounced in females and individuals under 75 years of age [10]. This was comparable with our findings.

The underlying mechanisms linking social frailty with adverse cognitive outcomes remain unclear but may be explained by factors such as social participation, social networks, and social isolation, all of which are components of social frailty. All the components of social frailty are associated with elevated inflammation levels, which correlate with cognitive decline and dementia [10,19,20]. Furthermore, social networks influence certain markers of Alzheimer's disease pathology [42]. Cognitive stimulation from social networks and activities improves the cognitive reserve and enhances cognitive function [42,43]. On the contrary, limited social relationships and networks may hinder individuals from accessing beneficial health information and increase the risk of developing unhealthy behavioral and psychological patterns that accelerate cognitive decline [15,44]. Frequent social interactions and active participation in social activities decrease stress by attenuating physiological responses, including activation of the hypothalamic-pituitary-adrenal (HPA) axis [15].

Education plays a crucial role in enhancing cognitive reserve, which protects against Alzheimer's disease-related pathological changes in the brain [42]. In this study, education was also considered as an indicator of social frailty. Older women in China have significantly lower education than older men because of traditional societal norms [45]. Moreover, a study by Duan et al. [46] suggested that women were more affected by social isolation than men. These findings offer a potential explanation for the higher prevalence of adverse cognitive outcomes in socially frail women.

Individuals under the age of 80, particularly those who have recently retired or are in the early stages of aging, tend to have higher expectations of their abilities. They are more accustomed to participating in social activities, strengthening social network connections, and may still bear significant social roles and responsibilities. However, the weakening of social networks and roles because of social frailty negatively impacts younger older populations, leading to increased stress and a decline in self-rated health [47]. As a result, these individuals are more likely to experience feelings of depression and anxiety, which, in turn, negatively impact cognitive function [[47], [48], [49], [50], [51]].

This study highlighted the important role of psychological resilience in the relationship between social frailty and cognitive function. A previous study reported that psychological resilience acts as a mediator of the link between social support and cognitive function in older individuals [24]. This finding was consistent with our results. Furthermore, we found that the mediating function of psychological resilience varied by sex. The previous study showed that resilience mediated the relationship between social support and cognitive function in both men and women [24]. However, our data showed the mediating role of psychological resilience only in males. This may be because of several factors. Compared to women, older Chinese men tend to have higher education levels, which contribute to a broader knowledge base, greater access to health information and social resources, and more effective coping strategies [38]. These factors may enhance psychological resilience [52] and mitigate the impact of social frailty on cognitive function. From a traditional perspective, men are expected to provide financially while women manage household affairs. Men often shoulder a greater share of the financial burden. Therefore, men develop stronger coping mechanisms of stress and psychological resilience as they navigate their financial responsibilities and challenges [24,52]. Females tend to receive greater emotional support than males and are more likely to proactively seek out assistance from their social networks, behaviors that contribute to enhanced psychological resilience [24]. However, because of social frailty, females may struggle to maintain or improve their psychological resilience through these channels. In this study, women demonstrated lower levels of physical activity and reported poorer self-reported health, thereby further weakening their psychological resilience and reducing its protective effect in later life [53,54]. RCS analysis demonstrated that the odds ratios of adverse cognitive outcomes in males initially increased with higher social frailty scores but eventually plateaued or declined. This phenomenon may be partially ascribed to the mediating effect of psychological resilience in males, which mitigates or reduces the negative effects of social frailty.

This study found that the mediating effect was present only in the more advanced group. According to a Korean study [55], older men exhibited a stronger positive relationship between psychological resilience and cognitive performance compared to younger men. As individuals age, they encounter a higher frequency of adverse life events. Psychological resilience is a dynamic concept that can be enhanced in response to negative events experienced by older adults [50,56]. In advanced old age, individuals may experience reduced social support because of significant transitions in social roles and adverse events. Under these conditions, psychological resilience plays a prominent role in maintaining cognitive function.

Although the existing design that did not adjust for years of education has been accepted in previous studies [57,58], we performed additional analyses by including years of education (as a continuous variable) as a covariate in the model (Tables A13-A16 and Figures A3-A4 in Appendix 1). The results demonstrated that while the effect sizes of the main findings were attenuated, they remained robust. Interestingly, in the subgroup analysis, the effect size linking social frailty to cognitive impairment was significantly reduced among women, even falling below that observed for men. This suggested that education played a key role in shaping the components of social frailty among women. Older women tend to have lower education levels. This increases the risk of social frailty and impacts cognitive reserve, thereby impairing cognitive function through both direct and indirect effects. The influences of education-related components within the social frailty indicators can be reduced by adjusting for education as a covariate. Furthermore, after adjusting for education, the relationship between social frailty and cognitive impairment among individuals aged <80 years became a linear association rather than a complex nonlinear association. However, it remained marginally nonlinearly significant (0.05 <*P* < 0.1). This suggested that the cognitive reserve and social resources accumulated through education may partially buffer the cognitive damage caused by social frailty.

The current study has several drawbacks. First, future research studies should use more rigorous and multidimensional scales to examine the connections between social frailty and cognitive performance. Furthermore, subsequent studies should investigate the role of education in the relationship between social frailty and cognitive function in greater depth, considering potential differences based on sex and age. Second, cognitive performance and social frailty were assessed using self-reported data, which may be subject to recall bias. Third, even after controlling for known confounders, some possible confounders may remain unaccounted for. Fourth, previous studies have suggested a stronger association between social frailty and executive function, and their combined effects are significantly linked to geriatric syndromes, living space, and quality of life [59]. However, the MMSE used in this study primarily assessed global cognitive function and focused on memory and orientation. This may have underestimated the influence of social frailty on executive function and other cognitive domains. Moreover, the joint effects of social frailty and cognitive function on various health outcomes have not been explored. Fifth, the follow-up period was short and may be insufficient to fully capture the cumulative impact of social frailty on cognitive function because effects of physiological responses such as chronic stress and inflammatory reactions may need a longer time to manifest.

## 5 Conclusion

Our data suggested an association between social frailty, psychological resilience, and cognitive outcomes. Improving social frailty and fostering psychological resilience could be meaningful for maintaining cognitive function among older adults. Furthermore, sex and age differences among older adults should be taken into account when interpreting these associations.

## CRediT authorship contribution statement

Yun-jing Zhang: Conceptualization, Formal analysis, Writing-original draft, Writing-review & editing. Cong Zhang: Data curation, Writing-original draft, Writing-review & editing, Visualization. Qi-yuan Lyu: Conceptualization, Supervision, Funding acquisition, Writing-review & editing.

## Ethics approval

The CLHLS was approved by the Biomedical Ethics Committee of Peking University (IRB00001052-13074), This study was based on a secondary analysis of the CLHLS and therefore ethical approval was not required.

## Declaration of Generative AI and AI-assisted technologies in the writing process

While preparing this work, the authors used ChatGPT to improve language. After using this tool, the authors reviewed and edited the content as needed and took full responsibility for the content of the publication.

## Funding

This work is supported by National Natural Science Foundation of China (NO. 72274078).

## Availability of data and materials

All data generated and/or analyzed during this study are available at https://opendata.pku.edu.cn/. Due to partial missingness in the original data, the exact dataset used and/or analyzed during this study is available from the corresponding author [Qi-yuan Lyu/yuanqlv@163.com] on reasonable request.

## Declaration of competing interest

The authors declare that they have no known competing financial interests or personal relationships that could have appeared to influence the work reported in this paper.
