# Supplementary material for: The association between social frailty, psychological resilience, and subsequent cognitive outcomes in older adults: A prospective cohort study
Source: J Nutr Health Aging. 2025 May 6;29(7):100576. doi: 10.1016/j.jnha.2025.100576 (PMC12172973; doi:10.1016/j.jnha.2025.100576)
Supplement: Supplementary file 1 [file mmc1.docx]

***Supplementary Material***

**Figure A1**

The flow chart of the inclusion of Chinese elderly.

**
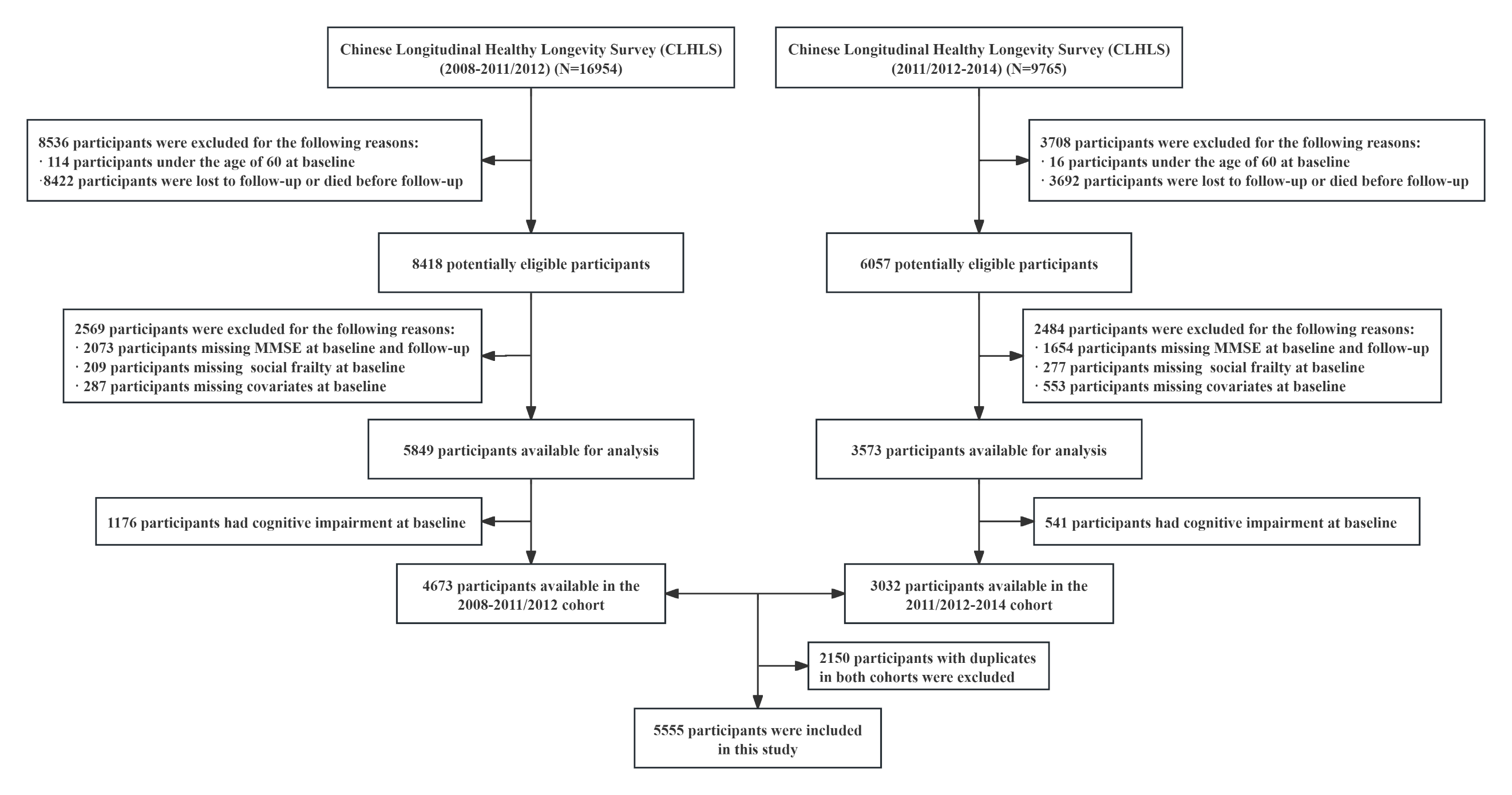
**

**Figure A2**

The distribution of the six variables used in this study within the theoretical framework proposed by Bunt.

**
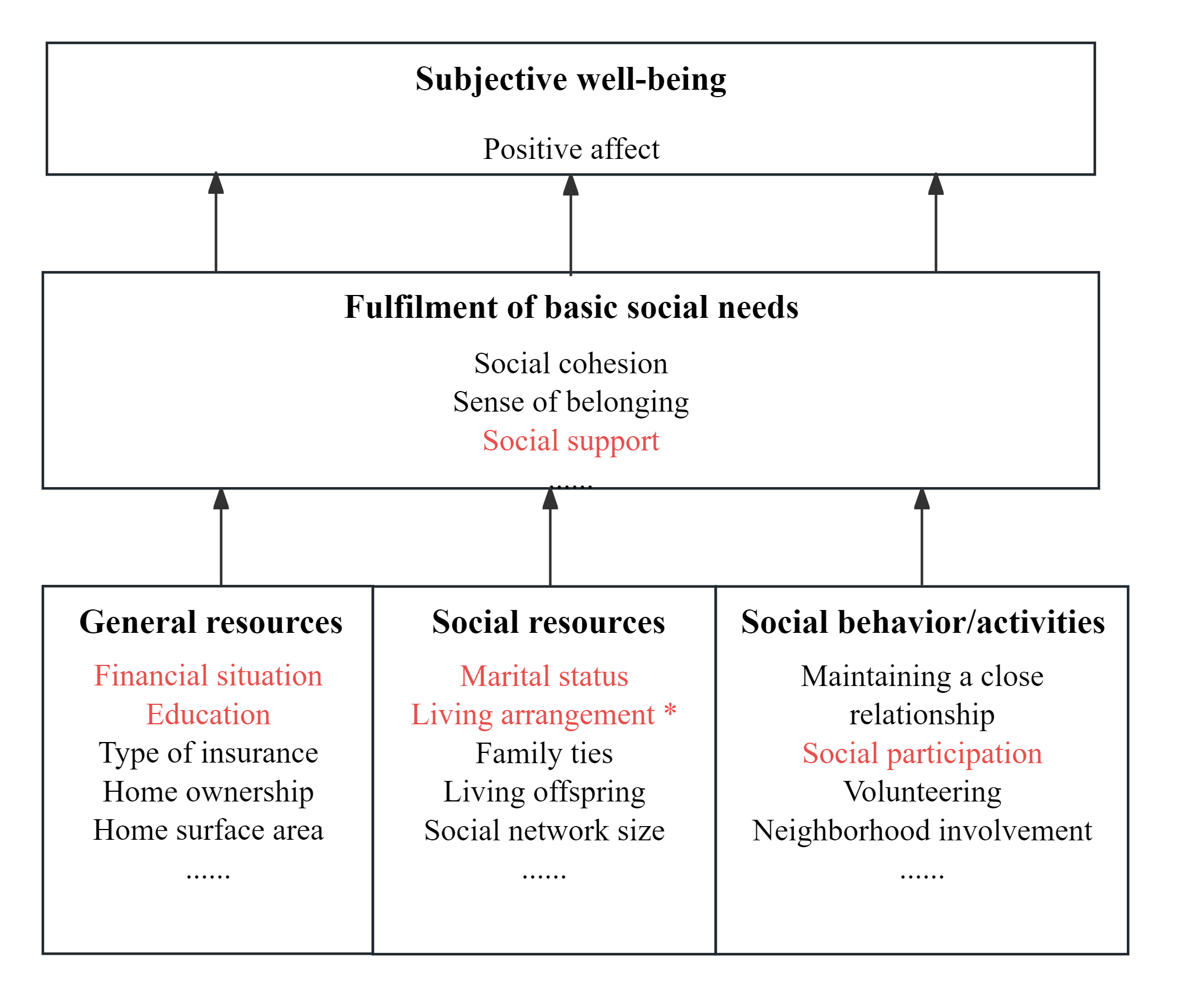
**

* Consistent with Yamada et al. [1], living arrangement has been incorporated as part of the social resource.

The indicators marked in red in the figure represent the measurement variables used in the present study.

**Table A1**

The distribution of the six variables used in this study within the theoretical framework proposed by Bunt.

| **Bunt's concept of social frailty** | **Our study** |
| --- | --- |
| General resources | Economic status |
|  | Education |
| Social resources | Marital status |
|  | Living arrangement ^*^ |
| Social behaviors or activities | Social participation   1. Playing mahjong 2. Participating in organized social activities 3. Going on outings |
| Needs fulfillment | Social support  (1) Who do you talk to most often?  (2) If you have something on your mind, who do you speak to?  (3) If you face a problem or difficulty, who do you seek help from?  (4) Currently, who mainly cares for you when you are unwell or sick? |

* Consistent with Yamada et al. [1], living arrangement has been incorporated as part of the social resource.

**Table A2**

Correlation of social frailty, psychological resilience, and cognitive function.

|  | **Social frailty score** | **Psychological resilience score** | | **MMSE score** |
| --- | --- | --- | --- | --- |
| **Social frailty score** | 1 | |  |  |
| **Psychological resilience score** | -0.293^⁎⁎⁎^ | | 1 |  |
| **MMSE score** | -0.339^⁎⁎⁎^ | | 0.165^⁎⁎⁎^ | 1 |

⁎⁎⁎ *p < 0.001*

**Table A3**

Mediating effect of psychological resilience on relationships between social frailty and cognitive function.

| **Step** | **Independent variable** | **Dependent variable** | **β** | **SE** | **T** | ***P*** | **95%CI** | **R** | **R-sq** |
| --- | --- | --- | --- | --- | --- | --- | --- | --- | --- |
| **Total** | | | | | | | | | |
| 1 | Social frailty | Cognitive function | -0.1308 | 0.0143 | -9.1615 | < 0.0001 | (-0.1587, -0.1028) | 0.4206 | 0.1769 |
| 2 | Social frailty | Psychological resilience | -0.1732 | 0.0141 | -12.2685 | < 0.0001 | (-0.2009, -0.1455) | 0.4415 | 0.1950 |
| 3 | Social frailty | Cognitive function | -0.1244 | 0.0145 | -8.6045 | < 0.0001 | (-0.1527, -0.0961) | 0.4219 | 0.1780 |
|  | Psychological resilience |  | 0.0367 | 0.0136 | 2.7039 | 0.0069 | (0.0101, 0.0634) |  |  |
| **Males** | | | | | | | | | |
| 1 | Social frailty | Cognitive function | -0.1221 | 0.0191 | -6.4043 | < 0.0001 | (-0.1594, -0.0847) | 0.3631 | 0.1318 |
| 2 | Social frailty | Psychological resilience | -0.1724 | 0.0186 | -9.2804 | < 0.0001 | (-0.2089, -0.1360) | 0.4180 | 0.1747 |
| 3 | Social frailty | Cognitive function | -0.1130 | 0.0193 | -5.8496 | < 0.0001 | (-0.1509, -0.0751) | 0.3662 | 0.1341 |
|  | Psychological resilience |  | 0.0525 | 0.0191 | 2.7523 | 0.0060 | (0.0151, 0.0899) |  |  |
| **Females** | | | | | | | | | |
| 1 | Social frailty | Cognitive function | -0.1260 | 0.0195 | -6.4729 | < 0.0001 | (-0.1641, -0.0878) | 0.4211 | 0.1773 |
| 2 | Social frailty | Psychological resilience | -0.1597 | 0.0194 | -8.2314 | < 0.0001 | (-0.1978, -0.1217) | 0.4264 | 0.1818 |
| 3 | Social frailty | Cognitive function | -0.1211 | 0.0197 | -6.1470 | < 0.0001 | (-0.1597, -0.0825) | 0.4220 | 0.1781 |
|  | Psychological resilience |  | 0.0303 | 0.0196 | 1.5492 | 0.1214 | (-0.0081, 0.0687) |  |  |
| **< 80 y** | | | | | | | | | |
| 1 | Social frailty | Cognitive function | -0.1617 | 0.0193 | -8.3826 | < 0.0001 | (-0.1995, -.01239) | 0.2680 | 0.0718 |
| 2 | Social frailty | Psychological resilience | -0.1862 | 0.0181 | -10.3119 | < 0.0001 | (-0.2216, -0.1508) | 0.4324 | 0.1869 |
| 3 | Social frailty | Cognitive function | -0.1599 | 0.0196 | -8.1507 | < 0.0001 | (-0.1984, -0.1215) | 0.2682 | 0.0719 |
|  | Psychological resilience |  | 0.0095 | 0.0193 | 0.4920 | 0.6228 | (-0.0283, 0.0472) |  |  |
| **≥ 80 y** | | | | | | | | | |
| 1 | Social frailty | Cognitive function | -0.1289 | 0.0219 | -5.8849 | < 0.0001 | (-0.1718, -0.0859) | 0.3114 | 0.0970 |
| 2 | Social frailty | Psychological resilience | -0.1455 | 0.0207 | -7.0200 | < 0.0001 | (-0.1862, -0.1049) | 0.4372 | 0.1911 |
| 3 | Social frailty | Cognitive function | -0.1202 | 0.0221 | -5.4408 | < 0.0001 | (-0.1635, -0.0769) | 0.3160 | 0.0999 |
|  | Psychological resilience |  | 0.0597 | 0.0214 | 2.7932 | 0.0053 | (0.0178, 0.1017) |  |  |

All models adjusted for age, sex, residence, smoking, drinking, exercise, BMI, ADL, sleep quality, self-reported health, MMSE score at baseline, hypertension, diabetes, and cerebrovascular disease.

**Table A4**

The joint associations between social frailty and psychological resilience with adverse cognitive outcomes.

| **Factor** | **OR (95 % CI)** |
| --- | --- |
| **Cognitive impairment** | |
| **Robust** | |
| High psychological resilience | Ref. |
| Low psychological resilience | 1.53 (1.10, 2.11) |
| **Social pre-frailty** | |
| High psychological resilience | 2.35 (1.73, 3.21) |
| Low psychological resilience | 2.39 (1.79, 3.20) |
| **Social frailty** | |
| High psychological resilience | 2.25 (1.41, 3.59) |
| Low psychological resilience | 3.65 (2.61, 5.10) |
|  | |
| **Greater cognitive decline** | |
| **Robust** | |
| High psychological resilience | Ref. |
| Low psychological resilience | 1.51 (1.20, 1.91) |
| **Social pre-frailty** | |
| High psychological resilience | 2.30 (1.82, 2.91) |
| Low psychological resilience | 2.15 (1.73, 2.68) |
| **Social frailty** | |
| High psychological resilience | 2.02 (1.36, 3.01) |
| Low psychological resilience | 3.05 (2.33, 4.00) |

All models adjusted for age, sex, residence, smoking, drinking, exercise, BMI, ADL, sleep quality, self-reported health, MMSE score at baseline, hypertension, diabetes, and cerebrovascular disease.

**Table A5**

Sensitivity analysis by using z-scores in BMI, MMSE score at baseline, and psychological resilience scores.

| **Outcome** | **Robust** | **Social pre-frailty** | **Social frailty** | ***P* for trend** |
| --- | --- | --- | --- | --- |
| **Total** | | | |  |
| Cognitive impairment | Ref. | 1.82 (1.49, 2.22) | 2.40 (1.86, 3.09) | <0.001 |
| Greater cognitive decline | Ref. | 1.72 (1.47, 2.01) | 2.09 (1.69, 2.60) | <0.001 |
| **Sex** | | | |  |
| **Males** | | | |  |
| Cognitive impairment | Ref. | 1.78 (1.37, 2.31) | 2.18 (1.44, 3.32) | <0.001 |
| Greater cognitive decline | Ref. | 1.72 (1.40, 2.10) | 1.51 (1.04, 2.18) | <0.001 |
| **Females** | | | |  |
| Cognitive impairment | Ref. | 1.89 (1.38, 2.58) | 2.50 (1.76, 3.57) | <0.001 |
| Greater cognitive decline | Ref. | 1.80 (1.41, 2.31) | 2.45 (1.82, 3.30) | <0.001 |
| **Age** | | | |  |
| **< 80 y** | | | |  |
| Cognitive impairment | Ref. | 2.32 (1.69, 3.18) | 3.42 (2.23, 5.26) | <0.001 |
| Greater cognitive decline | Ref. | 1.96 (1.57, 2.45) | 2.56 (1.83, 3.58) | <0.001 |
| **≥ 80 y** | | | |  |
| Cognitive impairment | Ref. | 1.58 (1.22, 2.04) | 1.99 (1.45, 2.74) | <0.001 |
| Greater cognitive decline | Ref. | 1.55 (1.24, 1.93) | 1.83 (1.37, 2.45) | <0.001 |

All models adjusted for age, sex, residence, smoking, drinking, exercise, BMI, ADL, sleep quality, self-reported health, psychological resilience score, MMSE score at baseline, hypertension, diabetes, and cerebrovascular disease

**Table A6**

Sensitivity analysis of the joint associations between social frailty and psychological resilience with adverse cognitive outcomes by using z-scores in BMI and MMSE score at baseline.

| **Factor** | **OR (95% CI)** |
| --- | --- |
| **Cognitive impairment** | |
| **Robust** | |
| High psychological resilience | Ref. |
| Low psychological resilience | 1.52 (1.10, 2.11) |
| **Social pre-frailty** | |
| High psychological resilience | 2.37 (1.74, 3.23) |
| Low psychological resilience | 2.41 (1.80, 3.20) |
| **Social frailty** | |
| High psychological resilience | 2.25 (1.41, 3.60) |
| Low psychological resilience | 3.65 (2.61, 5.10) |
|  | |
| **Greater cognitive decline** | |
| **Robust** | |
| High psychological resilience | Ref. |
| Low psychological resilience | 1.51 (1.20, 1.91) |
| **Social pre-frailty** | |
| High psychological resilience | 2.31 (1.83, 2.92) |
| Low psychological resilience | 2.17 (1.74, 2.69) |
| **Social frailty** | |
| High psychological resilience | 2.02 (1.35, 3.00) |
| Low psychological resilience | 3.06 (2.33, 4.00) |

All models adjusted for age, sex, residence, smoking, drinking, exercise, BMI, ADL, sleep quality, self-reported health, MMSE score at baseline, hypertension, diabetes, and cerebrovascular disease.

**Table A7**

Sensitivity analysis by including vegetable and fruit intake frequency and self-rated life quality as additional covariates.

| **Outcome** | **Robust** | **Social pre-frailty** | **Social frailty** | ***P* for trend** |
| --- | --- | --- | --- | --- |
| **Total** | | | |  |
| Cognitive impairment | Ref. | 1.83 (1.50, 2.23) | 2.42 (1.88, 3.12) | <0.001 |
| Greater cognitive decline | Ref. | 1.72 (1.47, 2.01) | 2.09 (1.69, 2.60) | <0.001 |
| **Sex** | | | |  |
| **Males** | | | |  |
| Cognitive impairment | Ref. | 1.80 (1.38, 2.34) | 2.29 (1.50, 3.48) | <0.001 |
| Greater cognitive decline | Ref. | 1.70 (1.39, 2.09) | 1.52 (1.05, 2.20) | <0.001 |
| **Females** | | | |  |
| Cognitive impairment | Ref. | 1.90 (1.39, 2.59) | 2.52 (1.77, 3.60) | <0.001 |
| Greater cognitive decline | Ref. | 1.80 (1.40, 2.31) | 2.45 (1.82, 3.31) | <0.001 |
| **Age** | | | |  |
| **< 80 y** | | | |  |
| Cognitive impairment | Ref. | 2.34 (1.70, 3.22) | 3.51 (2.28, 5.41) | <0.001 |
| Greater cognitive decline | Ref. | 1.96 (1.57, 2.45) | 2.59 (1.85, 3.62) | <0.001 |
| **≥ 80 y** | | | |  |
| Cognitive impairment | Ref. | 1.58 (1.23, 2.05) | 2.00 (1.46, 2.75) | <0.001 |
| Greater cognitive decline | Ref. | 1.54 (1.23, 1.92) | 1.82 (1.36, 2.43) | <0.001 |

All models adjusted for age, sex, residence, smoking, drinking, exercise, vegetable and fruit intake frequency, BMI, ADL, sleep quality, self-reported health, self-rated life quality, psychological resilience score, MMSE score at baseline, hypertension, diabetes, and cerebrovascular disease.

**Table A8**

Sensitivity analysis of the mediation effect by including vegetable and fruit intake frequency and self-rated life quality as additional covariates.

| **Effect** | **Effect value** | **SE** | **BootLLCI** | **BootULCI** |
| --- | --- | --- | --- | --- |
| **Total** | | | | |
| Total effect | -0.1324 | 0.0143 | -0.1605 | -0.1044 |
| Direct effect | -0.1260 | 0.0145 | -0.1544 | -0.0976 |
| Mediating effect | -0.0065 | 0.0025 | -0.0115 | -0.0017 |
| **Males** | | | | |
| Total effect | -0.1225 | 0.0191 | -0.1599 | -0.0851 |
| Direct effect | -0.1139 | 0.0193 | -0.1518 | -0.0760 |
| Mediating effect | -0.0086 | 0.0034 | -0.0154 | -0.0022 |
| **Females** | | | | |
| Total effect | -0.1290 | 0.0195 | -0.1672 | -0.0908 |
| Direct effect | -0.1238 | 0.0197 | -0.1625 | -0.0852 |
| Mediating effect | -0.0052 | 0.0034 | -0.0118 | 0.0013 |
| **< 80 y** |  |  |  |  |
| Total effect | -0.1638 | 0.0193 | -0.2017 | -0.1260 |
| Direct effect | -0.1623 | 0.0196 | -0.2008 | -0.1237 |
| Mediating effect | -0.0016 | 0.0040 | -0.0094 | 0.0064 |
| **≥ 80 y** | | | | |
| Total effect | -0.1311 | 0.0220 | -0.1742 | -0.0880 |
| Direct effect | -0.1220 | 0.0221 | -0.1654 | -0.0785 |
| Mediating effect | -0.0091 | 0.0034 | -0.0166 | -0.0032 |

All models adjusted for age, sex, residence, smoking, drinking, exercise, vegetable and fruit intake frequency, BMI, ADL, sleep quality, self-reported health, self-rated life quality, MMSE score at baseline, hypertension, diabetes, and cerebrovascular disease.

**Table A9**

Sensitivity analysis of the joint associations between social frailty and psychological resilience with adverse cognitive outcomes by including vegetable and fruit intake frequency and self-rated life quality as additional covariates

| **Factor** | **OR (95% CI)** |
| --- | --- |
| **Cognitive impairment** | |
| **Robust** | |
| High psychological resilience | Ref. |
| Low psychological resilience | 1.53 (1.10, 2.12) |
| **Social pre-frailty** | |
| High psychological resilience | 2.38 (1.74, 3.24) |
| Low psychological resilience | 2.44 (1.82, 3.26) |
| **Social frailty** | |
| High psychological resilience | 2.29 (1.43, 3.66) |
| Low psychological resilience | 3.70 (2.65, 5.18) |
|  | |
| **Greater cognitive decline** | |
| **Robust** | |
| High psychological resilience | Ref. |
| Low psychological resilience | 1.51 (1.20, 1.91) |
| **Social pre-frailty** | |
| High psychological resilience | 2.31 (1.83, 2.92) |
| Low psychological resilience | 2.16 (1.74, 2.69) |
| **Social frailty** | |
| High psychological resilience | 2.03 (1.36, 3.02) |
| Low psychological resilience | 3.05 (2.33, 4.00) |

All models adjusted for age, sex, residence, smoking, drinking, exercise, vegetable and fruit intake frequency, BMI, ADL, sleep quality, self-reported health, self-rated life quality, MMSE score at baseline, hypertension, diabetes, and cerebrovascular disease.

**Table A10**

Sensitivity analyses using multiple imputation

| **Outcome** | **Robust** | **Social pre-frailty** | **Social frailty** | ***P* for trend** |
| --- | --- | --- | --- | --- |
| **Total** | | | |  |
| Cognitive impairment | Ref. | 1.88 (1.54, 2.28) | 2.46 (1.92, 3.15) | <0.001 |
| Greater cognitive decline | Ref. | 1.75 (1.50, 2.03) | 2.14 (1.73, 2.65) | <0.001 |
| **Sex** | | | |  |
| **Males** | | | |  |
| Cognitive impairment | Ref. | 1.78 (1.38, 2.31) | 2.30 (1.54, 3.44) | <0.001 |
| Greater cognitive decline | Ref. | 1.72 (1.40, 2.10) | 1.57 (1.10, 2.23) | <0.001 |
| **Females** | | | |  |
| Cognitive impairment | Ref. | 2.02 (1.48, 2.76) | 2.61 (1.84, 3.72) | <0.001 |
| Greater cognitive decline | Ref. | 1.87 (1.46, 2.40) | 2.54 (1.89, 3.41) | <0.001 |
| **Age** | | | |  |
| **< 80 y** | | | |  |
| Cognitive impairment | Ref. | 2.44 (1.78, 3.34) | 3.48 (2.28, 5.32) | <0.001 |
| Greater cognitive decline | Ref. | 1.99 (1.60, 2.48) | 2.63 (1.89, 3.65) | <0.001 |
| **≥ 80 y** | | | |  |
| Cognitive impairment | Ref. | 1.60 (1.25, 2.06) | 2.04 (1.50, 2.78) | <0.001 |
| Greater cognitive decline | Ref. | 1.56 (1.26, 1.94) | 1.86 (1.41, 2.47) | <0.001 |

All models adjusted for age, sex, residence, smoking, drinking, exercise, BMI, ADL, sleep quality, self-reported health, psychological resilience score, MMSE score at baseline, hypertension, diabetes, and cerebrovascular disease.

**Table A11**

Sensitivity analysis of the mediation effect by using multiple imputation.

| **Effect** | **Effect value** | **SE** | **BootLLCI** | **BootULCI** |
| --- | --- | --- | --- | --- |
| **Total** |  |  |  |  |
| Total effect | -0.1312 | 0.0140 | -0.1586 | -0.1037 |
| Direct effect | -0.1249 | 0.0142 | -0.1527 | -0.0972 |
| Mediating effect | -0.0062 | 0.0023 | -0.0108 | -0.0020 |
| **Males** | | | | |
| Total effect | -0.1264 | 0.0188 | -0.1632 | -0.0897 |
| Direct effect | -0.1176 | 0.0190 | -0.1549 | -0.0804 |
| Mediating effect | -0.0088 | 0.0033 | -0.0158 | -0.0028 |
| **Females** | | | | |
| Total effect | -0.1242 | 0.0191 | -0.1617 | -0.0868 |
| Direct effect | -0.1195 | 0.0193 | -0.1574 | -0.0817 |
| Mediating effect | -0.0047 | 0.0031 | -0.0111 | 0.0010 |
| **< 80 y** | | | | |
| Total effect | -0.1617 | 0.0190 | -0.1989 | -0.1245 |
| Direct effect | -0.1596 | 0.0193 | -0.1974 | -0.1219 |
| Mediating effect | -0.0021 | 0.0036 | -0.0093 | 0.0049 |
| **≥ 80 y** | | | | |
| Total effect | -0.1290 | 0.0214 | -0.1711 | -0.0870 |
| Direct effect | -0.1207 | 0.0216 | -0.1631 | -0.0784 |
| Mediating effect | -0.0083 | 0.0032 | -0.0149 | -0.0026 |

All models adjusted for age, sex, residence, smoking, drinking, exercise, BMI, ADL, sleep quality, self-reported health, MMSE score at baseline, hypertension, diabetes, and cerebrovascular disease.

**Table A12**

Sensitivity analysis of the joint associations between social frailty and psychological resilience with adverse cognitive outcomes by using multiple imputation.

| **Factor** | **OR (95% CI)** |
| --- | --- |
| **Cognitive impairment** | |
| **Robust** | |
| High psychological resilience | Ref. |
| Low psychological resilience | 1.50 (1.09, 2.08) |
| **Social pre-frailty** | |
| High psychological resilience | 2.37 (1.74, 3.23) |
| Low psychological resilience | 2.49 (1.87, 3.33) |
| **Social frailty** | |
| High psychological resilience | 2.19 (1.38, 3.47) |
| Low psychological resilience | 3.77 (2.71, 5.24) |
|  | |
| **Greater cognitive decline** | |
| **Robust** | |
| High psychological resilience | Ref. |
| Low psychological resilience | 1.48 (1.17, 1.86) |
| **Social pre-frailty** | |
| High psychological resilience | 2.30 (1.82, 2.90) |
| Low psychological resilience | 2.18 (1.76, 2.70) |
| **Social frailty** | |
| High psychological resilience | 2.00 (1.35, 2.95) |
| Low psychological resilience | 3.09 (2.37, 4.04) |

All models adjusted for age, sex, residence, smoking, drinking, exercise, BMI, ADL, sleep quality, self-reported health, MMSE score at baseline, hypertension, diabetes, and cerebrovascular disease.

**Table A13**

Participants’ years of education at baseline.

| **Characteristic** | **Overall**  **(n=5555)** | **Robust**  **(n=2229)** | **Social pre-frailty**  **(n=2624)** | **Social frailty**  **(n=702)** | | ***P*** |
| --- | --- | --- | --- | --- | --- | --- |
| Years of education, median  [25th, 75th] | 1.00  [0.00, 5.00] | 5.00  [2.00, 8.00] | 0.00  [0.00, 3.00] | 0.00  [0.00, 0.00] | **<0.001** | |

*P*-values were calculated using rank sum tests.

**Table A14**

Association of social frailty with cognitive outcomes, including years of education.

| **Outcome** | **Robust** | **Social pre-frailty** | **Social frailty** | ***P* for trend** |
| --- | --- | --- | --- | --- |
| **Total** | | | |  |
| Cognitive impairment | Ref. | 1.56 (1.27 ,1.92) | 1.98 (1.52 ,2.57) | <0.001 |
| Greater cognitive decline | Ref. | 1.44 (1.22, 1.69) | 1.67 (1.33, 2.09) | <0.001 |
| **Sex** | | | |  |
| **Males** | | | |  |
| Cognitive impairment | Ref. | 1.65 (1.26, 2.17) | 1.96 (1.27, 3.02) | <0.001 |
| Greater cognitive decline | Ref. | 1.54 (1.25, 1.90) | 1.27 (0.87, 1.85) | 0.003 |
| **Females** | | | |  |
| Cognitive impairment | Ref. | 1.38 (0.99, 1.93) | 1.74 (1.19, 2.53) | 0.004 |
| Greater cognitive decline | Ref. | 1.30 (1.00, 1.70) | 1.67 (1.22, 2.30) | 0.001 |
| **Age** | | | |  |
| **< 80 y** | | | |  |
| Cognitive impairment | Ref. | 1.72 (1.23, 2.42) | 2.41 (1.54, 3.79) | <0.001 |
| Greater cognitive decline | Ref. | 1.48 (1.17, 1.88) | 1.82 (1.28, 2.59) | <0.001 |
| **≥ 80 y** | | | |  |
| Cognitive impairment | Ref. | 1.43 (1.10, 1.87) | 1.77 (1.27, 2.46) | 0.001 |
| Greater cognitive decline | Ref. | 1.39 (1.10, 1.74) | 1.59 (1.18, 2.15) | 0.002 |

All models adjusted for age, sex, residence, years of education, smoking, drinking, exercise, vegetable and fruit intake frequency, BMI, ADL, sleep quality, self-reported health, self-rated life quality, psychological resilience score, MMSE score at baseline, hypertension, diabetes, and cerebrovascular disease.

**Figure A3**

Restricted cubic spline regression model results show the association between social frailty and cognitive outcomes in the entire cohort as well as female and male subgroups, including years of education.


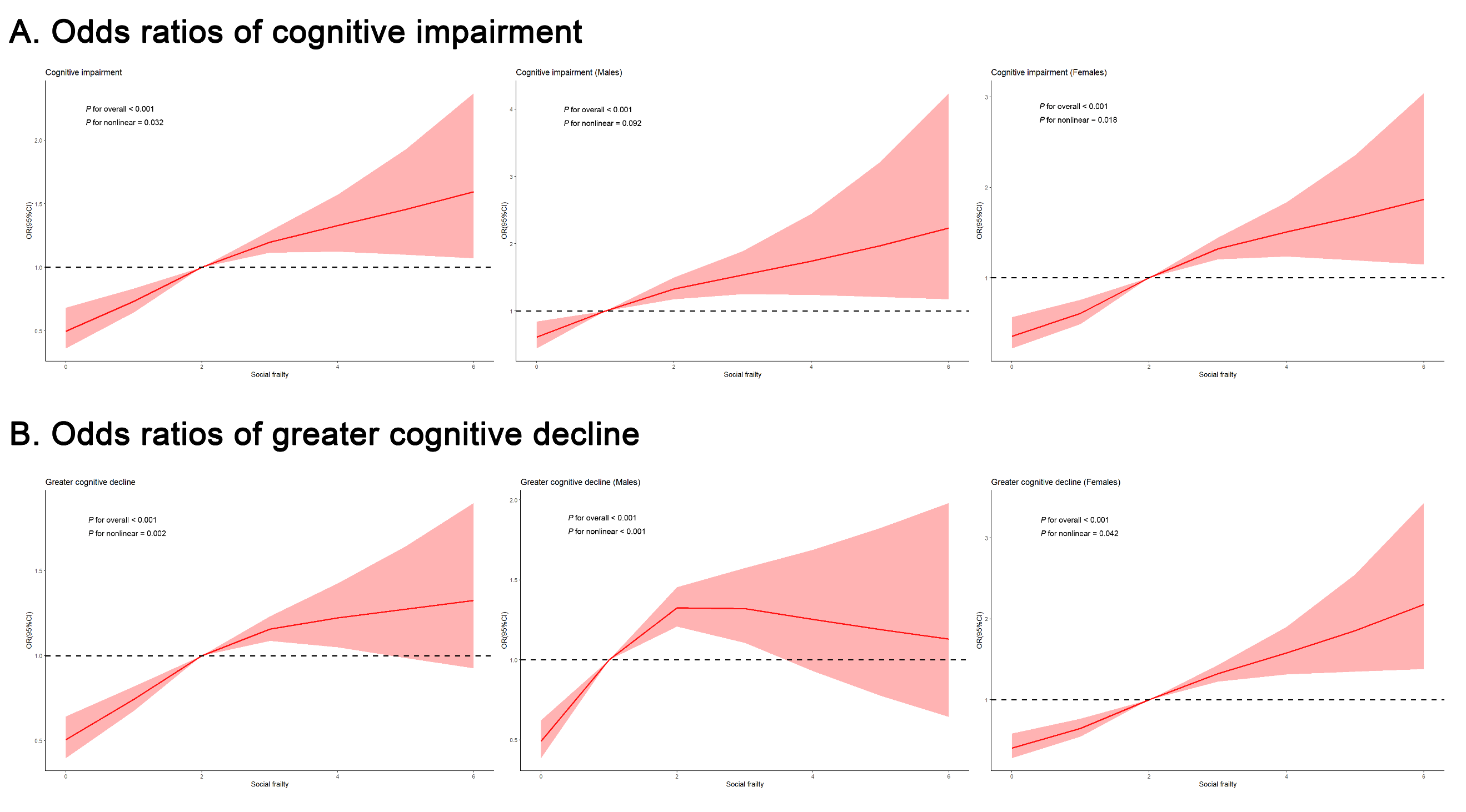


All models adjusted for age, sex, residence, years of education, smoking, drinking, exercise, BMI, ADL, sleep quality, self-reported health, psychological resilience score, MMSE score at baseline, hypertension, diabetes, and cerebrovascular disease.

**Figure A4**

Restricted cubic spline regression model results show the association between social frailty and cognitive outcomes in subjects belonging to >80 y and <80 y subgroups, including years of education.


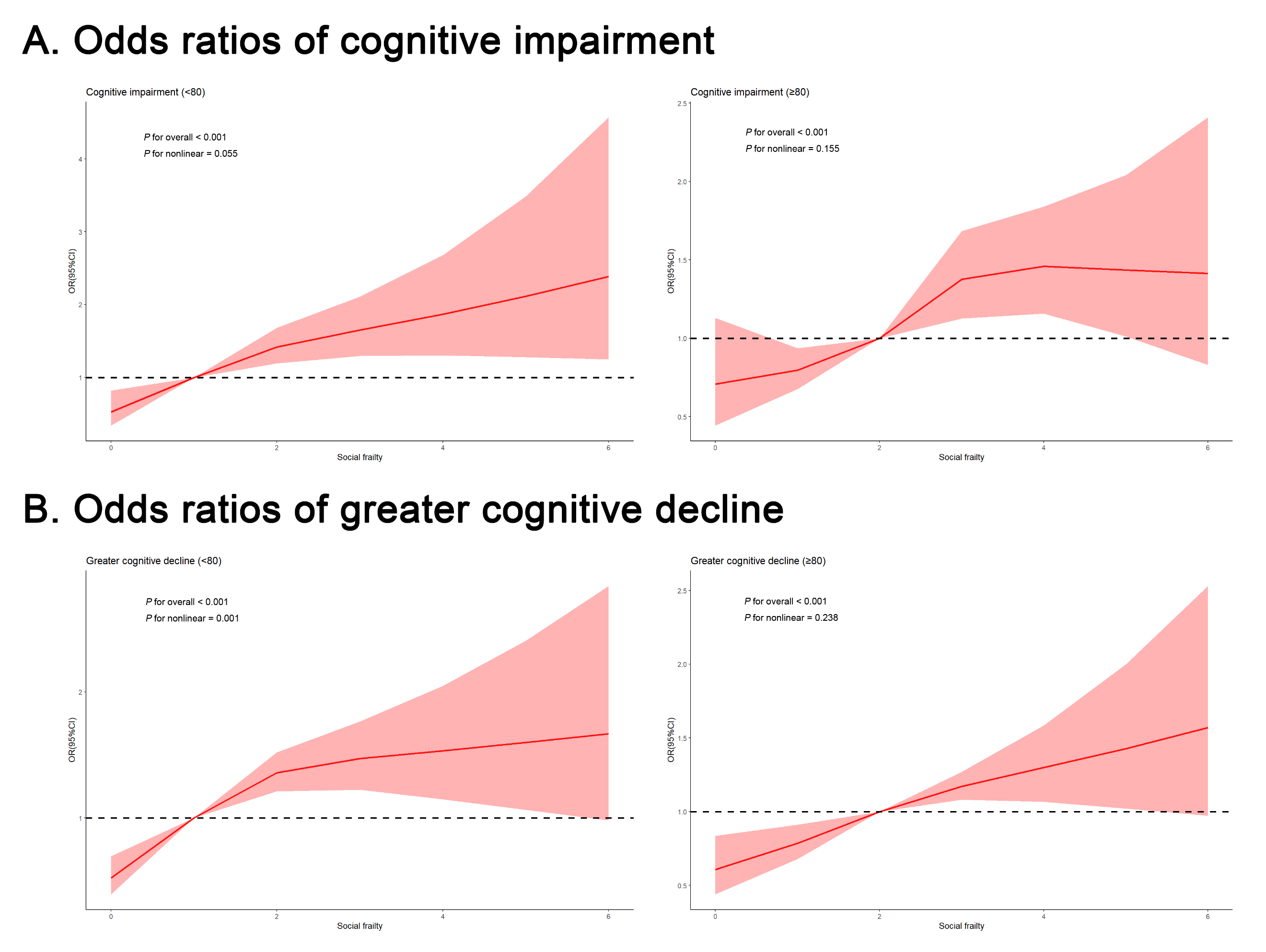


All models adjusted for age, sex, residence, years of education, smoking, drinking, exercise, BMI, ADL, sleep quality, self-reported health, psychological resilience score, MMSE score at baseline, hypertension, diabetes, and cerebrovascular disease.

**Table A15**

Mediation effect of psychological resilience on the association between social frailty and cognitive function, including years of education.

| **Effect** | **Effect value** | **SE** | **BootLLCI** | **BootULCI** |
| --- | --- | --- | --- | --- |
| **Total** | | | | |
| Total effect | -0.1313 | 0.0143 | -0.1593 | -0.1033 |
| Direct effect | -0.1249 | 0.0145 | -0.1533 | -0.0966 |
| Mediating effect | -0.0064 | 0.0025 | -0.0112 | -0.0017 |
| **Males** | | | | |
| Total effect | -0.1213 | 0.0190 | -0.1586 | -0.0840 |
| Direct effect | -0.1125 | 0.0193 | -0.1503 | -0.0747 |
| Mediating effect | -0.0088 | 0.0034 | -0.0158 | -0.0025 |
| **Females** | | | | |
| Total effect | -0.1260 | 0.0195 | -0.1642 | -0.0878 |
| Direct effect | -0.1211 | 0.0197 | -0.1598 | -0.0825 |
| Mediating effect | -0.0048 | 0.0033 | -0.0116 | 0.0014 |
| **< 80 y** | | | | |
| Total effect | -0.1132 | 0.0209 | -0.1542 | -0.0723 |
| Direct effect | -0.1128 | 0.0211 | -0.1542 | -0.0715 |
| Mediating effect | -0.0004 | 0.0034 | -0.0070 | 0.0063 |
| **≥ 80 y** | | | | |
| Total effect | -0.1083 | 0.0230 | -0.1534 | -0.0633 |
| Direct effect | -0.1005 | 0.0231 | -0.1458 | -0.0551 |
| Mediating effect | -0.0079 | 0.0032 | -0.0146 | -0.0023 |

Abbreviation: Effect value, standardized regression coefficient; SE, standard error; BootLLCI, bootstrapping lower limit confidence interval; BootULCI, bootstrapping upper limit confidence interval.

All models adjusted for age, sex, residence, years of education, smoking, drinking, exercise, BMI, ADL, sleep quality, self-reported health, MMSE score at baseline, hypertension, diabetes, and cerebrovascular disease.

**Table A16**

The joint associations between social frailty and psychological resilience with adverse cognitive outcomes, including years of education.

| **Factor** | **OR (95% CI)** |
| --- | --- |
| **Cognitive impairment** | |
| **Robust** | |
| High psychological resilience | Ref. |
| Low psychological resilience | 1.52 (1.10, 2.11) |
| **Social pre-frailty** | |
| High psychological resilience | 2.35 (1.72, 3.21) |
| Low psychological resilience | 2.40 (1.79, 3.21) |
| **Social frailty** | |
| High psychological resilience | 2.25 (1.41, 3.60) |
| Low psychological resilience | 3.65 (2.61, 5.10) |
|  | |
| **Greater cognitive decline** | |
| **Robust** | |
| High psychological resilience | Ref. |
| Low psychological resilience | 1.51 (1.20, 1.91) |
| **Social pre-frailty** | |
| High psychological resilience | 2.30 (1.82, 2.91) |
| Low psychological resilience | 2.15 (1.73, 2.68) |
| **Social frailty** | |
| High psychological resilience | 2.02 (1.36, 3.01) |
| Low psychological resilience | 3.06 (2.33, 4.00) |

All models adjusted for age, sex, residence, years of education, smoking, drinking, exercise, BMI, ADL, sleep quality, self-reported health, MMSE score at baseline, hypertension, diabetes, and cerebrovascular disease.

**References**

[1] Yamada M, Arai H. Social Frailty Predicts Incident Disability and Mortality Among Community-Dwelling Japanese Older Adults. Journal of the American Medical Directors Association 2018;19:1099–103, doi:http://dx.doi.org/10.1016/j.jamda.2018.09.013.
